# IFN-γ and TNF-α drive a *CXCL10+ CCL2+* macrophage phenotype expanded in severe COVID-19 lungs and inflammatory diseases with tissue inflammation

**DOI:** 10.1186/s13073-021-00881-3

**Published:** 2021-04-20

**Authors:** Fan Zhang, Joseph R. Mears, Lorien Shakib, Jessica I. Beynor, Sara Shanaj, Ilya Korsunsky, Aparna Nathan, Accelerating Medicines Partnership Rheumatoid Arthritis and Systemic Lupus Erythematosus (AMP RA/SLE) Consortium, Laura T. Donlin, Soumya Raychaudhuri

**Affiliations:** 1 Center for Data Sciences, Brigham and Women’s Hospital, Boston, MA 02115 USA; 2 Division of Genetics, Department of Medicine, Brigham and Women’s Hospital, Boston, MA 02115 USA; 3 Department of Biomedical Informatics, Harvard Medical School, Boston, MA 02115 USA; 4 Broad Institute of MIT and Harvard, Cambridge, MA 02142 USA; 5 Division of Rheumatology, Inflammation, and Immunity, Brigham and Women’s Hospital and Harvard Medical School, Boston, MA 02115 USA; 6 Graduate Program in Physiology, Biophysics and Systems Biology, Weill Cornell Graduate School of Medical Sciences, New York, NY 10065 USA; 7 Arthritis and Tissue Degeneration, Hospital for Special Surgery, New York, NY USA; 8 Arthritis Research UK Centre for Genetics and Genomics, Centre for Musculoskeletal Research, The University of Manchester, Manchester, UK

**Keywords:** Single-cell transcriptomics, Single-cell multi-disease tissue integration, COVID-19, Inflammatory diseases, Macrophage stimulation, Macrophage heterogeneity

## Abstract

**Background:**

Immunosuppressive and anti-cytokine treatment may have a protective effect for patients with COVID-19. Understanding the immune cell states shared between COVID-19 and other inflammatory diseases with established therapies may help nominate immunomodulatory therapies.

**Methods:**

To identify cellular phenotypes that may be shared across tissues affected by disparate inflammatory diseases, we developed a meta-analysis and integration pipeline that models and removes the effects of technology, tissue of origin, and donor that confound cell-type identification. Using this approach, we integrated > 300,000 single-cell transcriptomic profiles from COVID-19-affected lungs and tissues from healthy subjects and patients with five inflammatory diseases: rheumatoid arthritis (RA), Crohn’s disease (CD), ulcerative colitis (UC), systemic lupus erythematosus (SLE), and interstitial lung disease. We tested the association of shared immune states with severe/inflamed status compared to healthy control using mixed-effects modeling. To define environmental factors within these tissues that shape shared macrophage phenotypes, we stimulated human blood-derived macrophages with defined combinations of inflammatory factors, emphasizing in particular antiviral interferons IFN-beta (IFN-β) and IFN-gamma (IFN-γ), and pro-inflammatory cytokines such as TNF.

**Results:**

We built an immune cell reference consisting of > 300,000 single-cell profiles from 125 healthy or disease-affected donors from COVID-19 and five inflammatory diseases. We observed a *CXCL10+ CCL2+* inflammatory macrophage state that is shared and strikingly abundant in severe COVID-19 bronchoalveolar lavage samples, inflamed RA synovium, inflamed CD ileum, and UC colon. These cells exhibited a distinct arrangement of pro-inflammatory and interferon response genes, including elevated levels of *CXCL10*, *CXCL9*, *CCL2*, *CCL3*, *GBP1, STAT1*, and *IL1B*. Further, we found this macrophage phenotype is induced upon co-stimulation by IFN-γ and TNF-α.

**Conclusions:**

Our integrative analysis identified immune cell states shared across inflamed tissues affected by inflammatory diseases and COVID-19. Our study supports a key role for IFN-γ together with TNF-α in driving an abundant inflammatory macrophage phenotype in severe COVID-19-affected lungs, as well as inflamed RA synovium, CD ileum, and UC colon, which may be targeted by existing immunomodulatory therapies.

## Background

Tissue inflammation is a unifying feature across disparate diseases. While tissue- and disease-specific factors shape distinct inflammatory microenvironments, seemingly unrelated diseases can respond to the same therapy. For example, anti-tumor necrosis factor (TNF) therapies have revolutionized treatment for joint inflammation in autoimmune rheumatoid arthritis (RA) [1], while patients with intestinal inflammation due to Crohn’s disease (CD) and ulcerative colitis (UC), collectively known as inflammatory bowel disease (IBD), also respond to anti-TNF medications [2]. Here, we posit that the deconstruction of tissues to the level of individually characterized cells and subsequent integration of these cells from various types of inflamed tissues could provide a platform to identify shared pathologic features across diseases and provide rationale for repurposing medications in outwardly dissimilar conditions.

Recent studies have detailed features of local tissue inflammation and immune dysfunction in COVID-19 and related diseases caused by SARS and MERS coronaviruses [3]. Consensus is building that extensive unchecked inflammation involving so-called “cytokine storm” is a driver of severe late-stage disease. A single-cell study of bronchoalveolar lavage fluid (BALF) in intubated COVID-19 patients identified two inflammatory macrophage subsets—one characterized by *CCL2*, *CCL3*, and *CXCL10* expression and a second by *FCN1* and *S100A8*—as potential mediators of pathology in this late-stage disease [4]. The presence of these macrophage subsets in the lung correlated with elevated circulating cytokines and extensive damage to the lung and vascular tissue. Reports looking at peripheral blood from large numbers of COVID-19 patients have consistently documented lymphopenia (reduced lymphocyte frequency) paired with increased levels of CD14+ monocytes and inflammatory cytokines, such as IL1B, TNF-α, IFN-α, and IFN-γ [5–7]. These factors are ineffective in lowering viral load while possibly contributing to cytokine release syndrome (CRS) [7]. Together, these studies indicate the importance of uncovering the full extent of cell states present in COVID-19 patients including within affected tissues, and in particular among macrophages. Further, the extent to which these cell states are shared between COVID-19 and other inflammatory diseases and their disease association may further clarify disease mechanisms and precisely define therapeutic targets.

Macrophages are pervasive throughout the body and pivotal to tissue homeostasis, where they tailor their function to the parenchymal functions of each tissue type. In inflammation, tissue-resident macrophages and infiltrating monocytes are activated not only by factors from the unique tissue microenvironment, but also by disease-associating factors such as byproducts of deregulated tissue homeostasis, tissue damage, gene expression differences due to genetic variants, immune reactions, and in some cases, infecting pathogens. The unprecedented plasticity and robust reactivity of macrophages and monocytes generates a spectrum of phenotypes yet to be fully defined in human disease that mediate clearance of noxious elements, but in some cases, such as in cytokine storm, aggravate disease pathology. These phenotypes include a range of pro-inflammatory and anti-microbial states that secrete key cytokines (e.g., TNF and IL-1B) and chemokines (e.g., CXCL10 and CXCL11) and other functional states geared towards debris clearance, dampening inflammation, and tissue reconstruction, as well as a variety of intermediate states [8–11]. Meta-analysis of reactive macrophage phenotypes in inflamed tissues across diseases may further refine our understanding of the complexity of human macrophage functions, identifying subsets potentially shared across immune disorders, and thereby providing a promising route towards repurposing therapeutic strategies.

Single-cell RNA-seq (scRNA-seq) has provided an opportunity to interrogate inflamed tissues and identify expanded and potentially pathogenic immune cell types [12]. We recently defined a distinct *CD14+ IL1B+* pro-inflammatory macrophage population that is markedly expanded in RA compared to osteoarthritis (OA), a non-inflammatory disease [13, 14]. Likewise, scRNA-seq studies on inflamed colonic tissues have identified inflammatory macrophage and fibroblast phenotypes with high levels of Oncostatin M (OSM) signaling factors that are associated with resistance to anti-TNF therapies [15]. Only very recently, developments in computational methods have made it possible to meta-analyze an expansive number of cells across various tissue states, while mitigating experimental and cohort-specific artifacts [16–22], therein assessing shared and distinct cell states in disparate inflamed tissues.

To define the key shared immune cell compartments between inflammatory diseases with COVID-19, we meta-analyzed and integrated tissue-level single-cell profiles from five inflammatory diseases and COVID-19. We created an immune cell reference consisting of 307,084 single-cell profiles from 125 donor samples from RA synovium, systemic lupus erythematosus (SLE) kidney, UC colon, CD ileum, interstitial lung disease, and COVID-19 BALF. This single-cell reference represents comprehensive immune cell types from different disease tissues with different inflammation levels, which can be used to investigate inflammatory diseases and their connections with COVID-19 in terms of immune cell responses. Using our meta-dataset reference, we identified major immune cell lineages including macrophages, dendritic cells, T cells, B cells, NK cells, plasma cells, mast cells, and cycling lymphocytes. Among these, we found two inflammatory *CXCL10+ CCL2+* and *FCN1+* macrophage states that are shared between COVID-19 and several of the inflammatory diseases we analyzed. To understand the factors driving these phenotypes, we stimulated human blood-derived macrophages with eight different combinations of inflammatory disease-associated cytokines and tissue-associating stromal cells. We demonstrated that the *CXCL10+ CCL2+* macrophages from severe COVID-19 lungs share a transcriptional phenotype with macrophages stimulated by TNF-α plus IFN-γ. Notably, the other two conditions wherein these macrophages are most abundant are RA and CD. As patients with RA and CD show response to anti-TNF therapies, this finding supports the approach of identifying shared cellular states in unrelated inflamed tissues to define shared responses to medications. Furthermore, janus kinase (JAK) inhibitors have also proved effective in RA, presumably in large part through targeting IFN-γ responses [8, 23, 24]. Our data collectively support the potential efficacy of JAK inhibitors and anti-TNF therapies in inflammatory macrophage responses in COVID-19 due to cellular phenotype associations with select inflammatory tissue diseases already proven to respond to these medications.

## Methods

### Integration of scRNA-seq profiles from multiple datasets

#### scRNA-seq data collection, remapping, and aggregation

To build a multi-tissue immune cell reference, we obtained the raw FASTQ files and raw count matrices from the following publicly available scRNA-seq datasets: RA synovial cells from dbGaP (Zhang, et al, 2019; phs001457.v1.p1) [13] and dbGaP (Stephenson, et al, 2018; phs001529.v1.p1) [25], SLE kidney cells from dbGaP (Arazi, et al, 2019; phs001457.v1.p1) [26], UC colon cells from Single Cell Portal (Smillie, et al, 2019; SCP259) [15], CD ileum cells from GEO (Martin, et al, 2019; GSE134809) [27], interstitial and pulmonary lung disease from GEO (Reyfman, et al, 2019; GSE122960) [28], and COVID-19 and healthy BALF cells from GEO (Liao, et al, 2020; GSE145926) [4]. We also use the datasets from Grant et al. (GSE155249) [29] and Xue et al. (GSE47189) [11] as additional validations.

For the FASTQs that we obtained, we used Kallisto [30] to map the raw reads to the same kallisto index generated from GRCh38 Ensembl v100 FASTA files. We pseudo-aligned FASTQ files to this reference, corrected barcodes, sorted BUS files, and counted unique molecular identifiers (UMIs) to generate UMI-count matrices. We aggregated all the cell barcodes from 125 donor samples into one matrix. We performed consistent QC to remove the cells that expressed fewer than 500 genes or with more than 20% of the number of UMIs mapping to the mitochondrial genes, resulting in 307,084 cells in total. The number of donor samples and cells that passed QC for each tissue source, disease status, technology, and clinical data are shown in Additional file 1: Table S1.

#### Normalization, scaling, and feature selection

We aggregated all samples on the overlapped 17,054 genes. We then normalized each cell to 10,000 reads and log-transformed the normalized data. We then selected the top 1,000 most highly variable genes based on dispersion within each donor sample and combined these genes to form a variable gene set. Based on the pooled highly variable genes, we then scaled the aggregated data matrix to have mean 0 and variance 1. We normalized the expression matrix using the L2 norm.

#### Dimensionality reduction and batch effect correction

To minimize the effect from multiple datasets with different cell numbers during an unbiased scRNA-seq data integration, we performed weighted principal component analysis (PCA) and used the first 20 weighted PCs for follow-up analysis. The summation of the weights for cells from each separate single-cell dataset is equal so that each dataset contributed equally to the analysis. For all cell-type integration, we corrected batch effects on three different levels (sequencing technology, tissue source, and donor sample) simultaneously using Harmony [16]. We use default parameters and also specified theta = 2 for each batch variable, max.iter.cluster = 30, and max.iter.harmony = 20. For Harmony batch correction, we use the same weights from the weighted PCA. For macrophage only integration, we corrected the effect from donors for the 10X data, and dataset for the CEL-seq2 data since each donor generated from CEL-seq2 data only has less than 100 cells. As outputs, we obtained batch-corrected PC embeddings where the effects from different single-cell datasets and donors are removed in low-dimensional PC space.

#### Quantitative evaluation of batch correction and dataset integration

Variance explained from different sources: To quantitatively measure the mixture of batch effects after correction, we estimated the sources of variance explained from gene expression on the first ten principal component embeddings. We show the proportion of variance explained from the original pre-defined immune cell type, tissue origin, technology, and donor sample. We used the R package limma [31] to fit the model and ANOVA to compute the percentage of variance explained:

$$ \mathrm{principal}\ \mathrm{component}\sim \mathrm{celltype}+\mathrm{tissue}+\mathrm{technology}+\mathrm{sample}. $$

LISI score: Meanwhile, we used a LISI (local inverse Simpson’s Index) metric to measure the mixture levels of batch labels based on local neighbors chosen at a specific perplexity [16, 22]. Specifically, we built Gaussian distribution of neighborhoods and computed these local distributions of batch probabilities *p*(*b*) using perplexity 30 on the first 20 principal components. *B* is the number of batches. Then, we calculated the inverse Simpson’s index:

$$ 1/{\sum}_{b=1}^Bp(b). $$

An iLISI (integration LISI) score ranges from 1.0, which denotes no mixing, to B (the maximum score is the total number of levels in the categorical batch variable) where higher scores indicate better mixing of batches. Here batch can be tissue source, donor sample, and sequencing technology. We also calculated the cLISI (cell-type LISI), which measures integration accuracy of pre-defined cell-type annotations instead of using the same formulation. An accurate embedding has a cLISI close to 1 for every cell neighborhood, reflecting separation of distinct cell types.

#### Graph-based clustering

We then applied unbiased graph-based clustering (Louvain [32]) on the top 20 batch-corrected PCs at various resolution levels (0.2, 0.4, 0.6, 0.8, 1.0). We use 0.4 as the final resolution value to gain the biological interpretations that make most sense. Then, we furthermore performed dimensionality reduction using UMAP [33].

#### Pseudo-bulk differential expression analysis

To identify robust single-cell cluster marker genes that are shared between diseases, we performed pseudo-bulk analysis by summing the raw UMI counts for each gene across cells from the same donor sample, tissue source, and cluster assignment. We modeled raw count as a negative binomial (NB) distribution and fitted a generalized linear model (GLM) for each gene accounting for tissue, sample, and nUMI using DESeq2 [34]. We also computed AUC and *P* using the Wilcoxon rank-sum test by comparing pseudo-bulk samples from one cluster to the others. We use several criteria to decide statistically significant marker genes: (1) GLM-β, (2) fold change, (3) AUC, and (4) Wilcoxon rank-sum test and Bonferroni-corrected *P* (threshold 10^−5^, 0.05/5,000 tested highly variable genes)*.* We tested all genes that were detected in more than 100 cells with non-zero UMI counts.

#### Identification of major immune cell-type clusters

We carefully annotated each identified immune cell-type cluster in two ways. First, we mapped the original published annotation labels [4, 13, 15, 26, 27] to our UMAP embeddings when applicable. We are able to reproduce the original cell-type subsets in our cross-disease integrative analysis. Second, we annotated the identified clusters using cell-type lineage marker genes: T cells (*CD3D*), NK cells (*NCAM1*), B cells (*MS4A1*), plasma cells (*MZB1*), macrophages (*FCGR3A/CD14*), dendritic cells (DCs, *CD1C*), mast cells (*TPSAB1*), and cycling cells (*MKI67*).

### Cell culture for human blood-derived macrophages and synovial fibroblasts

We obtained human leukocyte-enriched whole blood samples from 4 healthy blood donors from the New York Blood Center and purified peripheral blood mononuclear cells (PBMC) from each using Ficoll gradient centrifugation. We isolated CD14+ monocytes from each sample using human CD14 microbeads (Miltenyi Biotec) and differentiated these cells into blood-derived macrophages for 1 day at 37 °C in Macrophage-Colony Stimulating Factor (M­-CSF); 10 ng/mL) (PeproTech) and RPMI 1640 medium (Corning) supplemented with 10% defined fetal bovine serum (FBS) (HyClone), 1% penicillin-streptomycin (Thermo Fisher Scientific), and 1% l-glutamine (Thermo Fisher Scientific) in a 6-well plate at a concentration of 1.2 million cells/mL.

In parallel, we obtained human synovial fibroblasts derived from deidentified synovial tissues from RA patients undergoing arthroplasty (HSS IRB 14­033). Two unique fibroblast lines were used, each paired with two distinct blood-derived macrophage donor samples. We cultured fibroblasts in alpha minimum essential medium (a­MEM) (Gibco) supplemented with 10% premium FBS (R&D Systems Inc), 1% penicillin-streptomycin (Thermo Fisher Scientific), and 1% l-glutamine (Thermo Fisher Scientific) for 4 to 6 passages. To create each transwell, we seeded the mesh of polyester chambers with 0.4­μm pores (Corning) with either 200,000 synovial fibroblasts or without fibroblasts for 1 day at 37 °C.

The following day, we suspended each transwell—3 with fibroblasts and 6 without fibroblasts per donor—above one well of cultured macrophages. Those transwells with fibroblasts had a fibroblast-­to-­macrophage ratio of 1:15. In total, we created 9 wells per donor. Next, we added IFN-β (200 pg/mL), IL-4 (20 ng/ mL), TNF-α (20 ng/mL), and/or IFN-γ (5 ng/mL) to each transwell and underlying plate per donor. All plates were incubated at 37 °C for 19 h.

### RNA library preparation and sequencing

We applied a modified version of the staining protocol from CITE-seq, using only Totalseq™-A Hashing antibodies from Biolegend [35]. We harvested macrophages from each well and aliquoted one fifth of the cells, ~ 750,000 cells per condition, for staining in subsequent steps. We washed the cells in filtered labeling buffer (PBS with 1% BSA) and resuspended in 50 μL of labeling buffer with Human TruStain FcX™ (Biolegend Cat #422302, 5 μL per stain) for 10 min at 4 °C. Next, we added 50 μL of labeling buffer for a final concentration of 1.6 ng/μL of a total-seq hashtag (1, 2, 4–9, or 12) per condition per donor for 25 min at 4 °C. Next, we washed all samples in 2 mL, 1 mL, and 1 mL of labeling buffer, sequentially. We counted the remaining cells using a cellometer (Nexcelom Cellometer Auto 1000) and aliquoted the equivalent of 60,000 cells from each condition into one Eppendorf tube per donor. From here, we filtered through a 40-μm mesh and resuspended in PBS with 0.04% BSA to a concentration of 643.7 cells/μl. We followed the Chromium Single Cell 3′ v3 kit (10x Genomics) processing instructions and super-loaded 30,000 cells per lane. We used one lane per donor, with 9 conditions multiplexed per donor sample. After cDNA generation, samples were shipped to the Brigham and Women’s Hospital Single Cell Genomics Core for cDNA amplification and sequencing. Pairs of libraries were pooled and sequenced per lane on an Illumina NovaSeq S2 with paired-end 150 base-pair reads.

### Processing FASTQ reads into gene expression matrices and cell hashing

We quantified mRNA and antibody UMI counts, respectively. Cellranger v3.1.0 was used to process the raw BCL files and produce a final gene by cell barcode UMI count matrix. First, raw BCL files were demultiplexed using cellranger mkfastq to generate FASTQ files with default parameters. Then, these FASTQ files were aligned to the GRCh38 human reference genome. Gene/antibody reads were quantified simultaneously using cellranger count. Cell barcodes and UMIs were extracted for gene/hashtag antibodies for each run.

For quality control of the cells, we first performed mRNA-level cell QC and then hashtag-level QC. For the mRNA-level QC, we removed the cells that expressed fewer than 1,000 genes or more than 10% of UMIs mapping to the mitochondrial genes. For the hashtag QC, we removed the cells whose proportion of UMIs for the most abundant hashing antibody is less than 90%, and removed the cells whose ratio of the second most-abundant and first most-abundant antibody is greater than 0.10. After filtering, each cell was assigned a hashing antibody and donor sample on the most abundant hashing antibody barcode. After QC, we obtained 9,399, 8,775, 4,622, and 3,027 cells for the 4 donor samples. We then normalized UMI counts from each cell based on the total number of UMIs and log-transformed the normalized counts.

### Linear modeling for experimental stimulation-specific genes from cell culture single-cell profiles

To more accurately identify gene signatures that are specific to each of the eight stimulatory conditions, we used linear models to test each gene for differential normalized gene expression across contrasts of interest. Specifically, we fit the following models:

$$ \mathrm{gene}\_\mathrm{expression}\sim \mathrm{stim}+1\mid \mathrm{sample}+\mathrm{nUMI}, $$

where stim is a categorical variable that represents eight stimuli and an untreated status, 1 ∣ sample is the random effect of the 4 replicated donor samples, and nUMI (number of unique molecular identifiers) represents the technical cell-level fixed effect. We obtained the fold change, *T* and *P* value, and Bonferroni-corrected *P* value as measurements for each tested gene signature for each applied condition. We then generated a list of differentially expressed genes whose fold change is greater than 2 and *P* is smaller than the Bonferroni correction threshold 10^−7^ (0.05/7,000 highly variable genes × 9 conditions) for each stimulatory condition.

### Testing integrative macrophage clusters for association with severe/inflamed status

We tested the association of each macrophage cluster with severe/inflamed status compared to healthy with MASC (mixed-effects modeling of associations of single cells) [36]. We fit a logistic regression model for each identified cluster within one tissue and set the nUMIs and percent MT (% MT) content as cell-level fixed effects, and donor sample as a random effect:

$$ \log \left[\frac{Y_{i,c}}{1-{Y}_{i,c}}\right]={\beta}_{\mathrm{case}}{X}_{i,\mathrm{case}}+{\beta}_{\mathrm{tech}1}{X}_{i,\mathrm{tech}1}+{\beta}_{\mathrm{tech}2}{X}_{i,\mathrm{tech}2}+\left({\varphi}_d|\ d\ \right), $$

where *Y*_*i*,*c*_ is the odds of cell *i* in cluster *c*, *β*_case_ is the effect log (odds ratio) for case (severe COVID-19)-control (healthy) status, *β*_tech1_ is a vector of technical cell-level (nUMIs) covariate, *β*_tech2_ is a vector of technical cell-level (% mitochondrial genes) covariate, *X*_*i*_ is the values for cell *i* in technology as appropriate, and (*φ*_*d*_| *d* ) is the random effect of donor *d*. Thus, we used this logistic regression model to test for differentially abundant macrophage clusters associated with severe COVID-19 by correcting for the technical cell-level and donor-level covariates. Similarly, we also tested for differentially abundant macrophage clusters associated with inflamed CD compared to non-inflamed CD, RA compared to OA, and inflamed UC compared to healthy colon, accounting for technical cell-level and donor-specific covariates. We generated log likelihood-ratio test MASC *P* values and odds ratios for each tested cluster and used Bonferroni correction to report the macrophage clusters that are statistically significantly more abundant in severe/inflamed samples compared to healthy or non-inflamed controls.

### Gene score calculation

We calculated a *CXCL10+ CCL2+* gene score for each single-cell profile from an external single-cell RNA-seq dataset from severe COVID-19 BALF [29]. The gene score was calculated as the sum of counts for *CXCL10+ CCL2+* genes (*n* = ~ 70) as a percent of total gene counts for each cell.

### Pathway enrichment analysis

For pathway gene set enrichment, we use the msigdbr R package on 4872 genesets including C5 (Gene Ontology), C7 (immunologic signature), and H (Hallmarks) from MSigDB [37] to calculate enriched pathways of macrophage states for each disease tissue.

### Statistical analysis

For all the analysis and plots, sample sizes and measures of center and confidence intervals (mean ± SD or SEM), and statistical significance are presented in the figures, figure legends, and in the text. Results were considered statistically significant when *P* < 0.05 by Bonferroni correction as is indicated in figure legends and text.

## Results

### A reference of > 300,000 immune single-cell profiles across inflammatory diseases and COVID-19

**Fig. 1:**
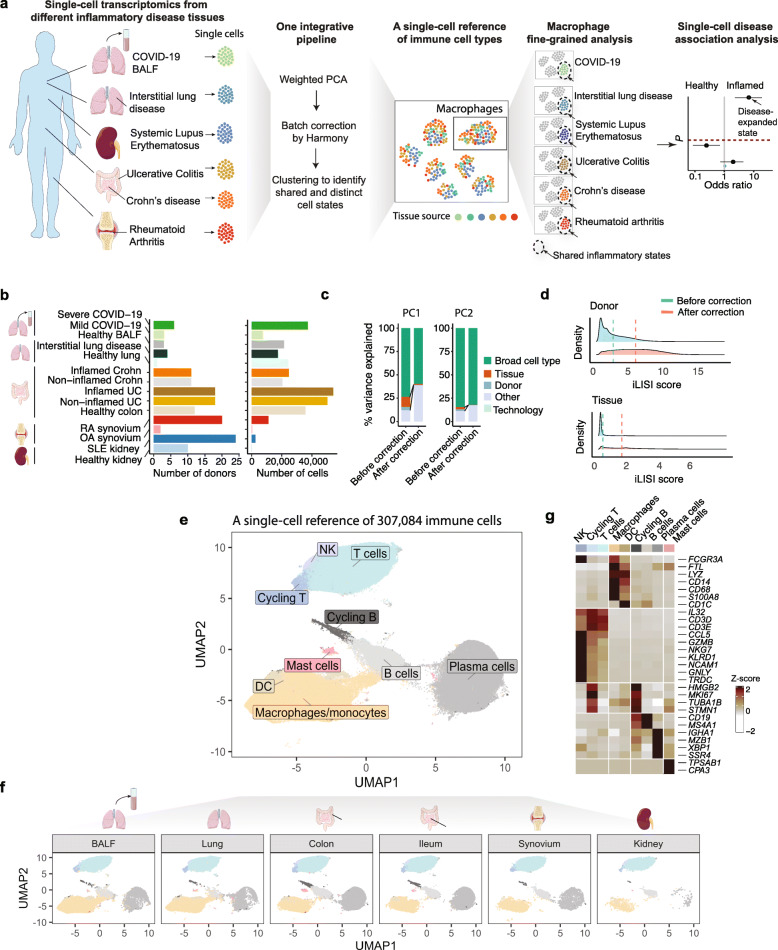
Integrative analysis of > 300,000 single-cell profiles from five inflammatory disease tissues and COVID-19 BALF. **a** Overall study design and single-cell analysis, including the integrative pipeline, a single-cell reference dataset, fine-grained analysis to identify shared macrophage states, and disease association analysis. **b** Number of cells and donor samples from each healthy and disease tissue. **c** Percent of variance explained in the gene expression data by pre-defined broad cell type, tissue, sample, and technology for the first and second principal component (PC1 and PC2) before and after batch effect correction. **d** iLISI score before and after batch correction to measure the mixing levels of donor samples and tissue sources. An iLISI (integration LISI) score of 1.0 denotes no mixing while higher scores indicate better mixing of batches. **e** Integrative clustering of 307,084 cells reveals common immune cell types from different tissue sources. **f** Immune cells from separate tissue sources in the same UMAP coordinates. Cells from the same cell types are projected next to each other in the integrative UMAP space. **g** Heatmap of cell-type lineage marker genes. Gene signatures were selected based on AUC > 0.6 and *P* < 0.05 by Bonferroni correction comparing cells from one cell type to the others

To compare hematopoietic cells across inflammatory diseases and COVID-19 in an unbiased fashion, we aggregated 307,084 single-cell RNA-seq profiles from 125 healthy or inflammatory disease-affected tissues spanning six disorders: (1) colon from healthy individuals and patients with inflamed or non-inflamed UC [15]; (2) terminal ileum from patients with inflamed or non-inflamed CD [27]; (3) synovium from patients with RA or OA [13, 25]; (4) kidney from patients with SLE or healthy controls [26], (5) lung from patients with interstitial lung disease [28], and (6) BALF from healthy individuals and those with mild or severe COVID-19 [4] (Fig. 1a, b, Additional file 2: Figure S1a, Additional file 1: Table S1). We developed a pipeline for multi-tissue integration and disease association at the single-cell level (Fig. 1a, “Methods”). Where feasible, we obtained raw reads and re-mapped them to the GRCh38 genome assembly. We then aggregated raw counts for 17,054 shared genes across studies into a single matrix, performed consistent quality control (QC), library size normalization, and principal component analysis [38] (PCA) (“Methods”). To account for different cell numbers from different datasets, we performed weighted PCA, assigning higher weights to cells from datasets with a relatively small number of cells and vice versa. In the integrated PCA embedding, we modeled and removed the effects of technology, tissue, and donor with Harmony [16] to identify shared cell states across studies and diseases (“Methods”). Before Harmony, cells grouped primarily based on tissue source (Additional file 2: Figure S1b). After Harmony, < 1% of the variation explained by PC1 and PC2 was attributable to tissue source and sample, while > 60% was attributable to previously defined cell types (Fig. 1c). Importantly, rare pathogenic cell types within tissue, such as germinal center B cells in inflamed UC colon and age-associated B cells in RA synovium, were identifiable in the integrated space (Additional file 2: Figure S1c). We confirmed the degree of cross sample, tissue, technology, and cell-type mixing with an independent measure of single-cell integration: LISI [16, 22] (Local Inverse Simpson’s Index). An increased iLISI (integration LISI) score after batch correction compared to before batch correction indicates a better mixing of batches after correction (Fig. 1d and Additional file 2: Figure S2a).

In this integrated space, we performed graph-based clustering [32] and visualization with UMAP (Uniform Manifold Approximation and Projection) [33]. We identified 9 major cell-type clusters (Fig. 1e) present in all six tissues (Fig. 1f) and diseases (Additional file 2: Figure S2b). We labeled the clusters with canonical markers (Fig. 1g, Additional file 3: Table S2): *CD3D+* T cells, *NCAM1*+ NK cells, *MS4A1+* B cells, *MZB1+* plasma cells, *FCGR3A*+/*CD14+* macrophages, *CD1C+* dendritic cells (DCs), *TPSAB1+* mast cells, and *MKI67+* cycling T and B cells.

While the proportion of these immune populations differed substantially among tissues, macrophages represented a major component in each tissue (Additional file 2: Figure S2c). For example, samples obtained from lung tissues and BALF, whether from healthy controls or patients with ILD and COVID-19, contained the highest proportion of macrophages (74.8% of total hematopoietic cells) (Fig. 1f, Additional file 2: Figure S2c). In contrast, while RA synovium, SLE kidney, and CD ileum contained 9.4% macrophages, T lymphocytes comprised the majority of cells in these tissues (55.7%). The UC colon samples contained 8.3% macrophages, but had a distinctively high abundance of plasma cells (42.4%) (Additional file 2: Figure S2c).

### Identification of shared inflammatory macrophage states across inflammatory disease tissues and COVID-19 lungs

**Fig. 2:**
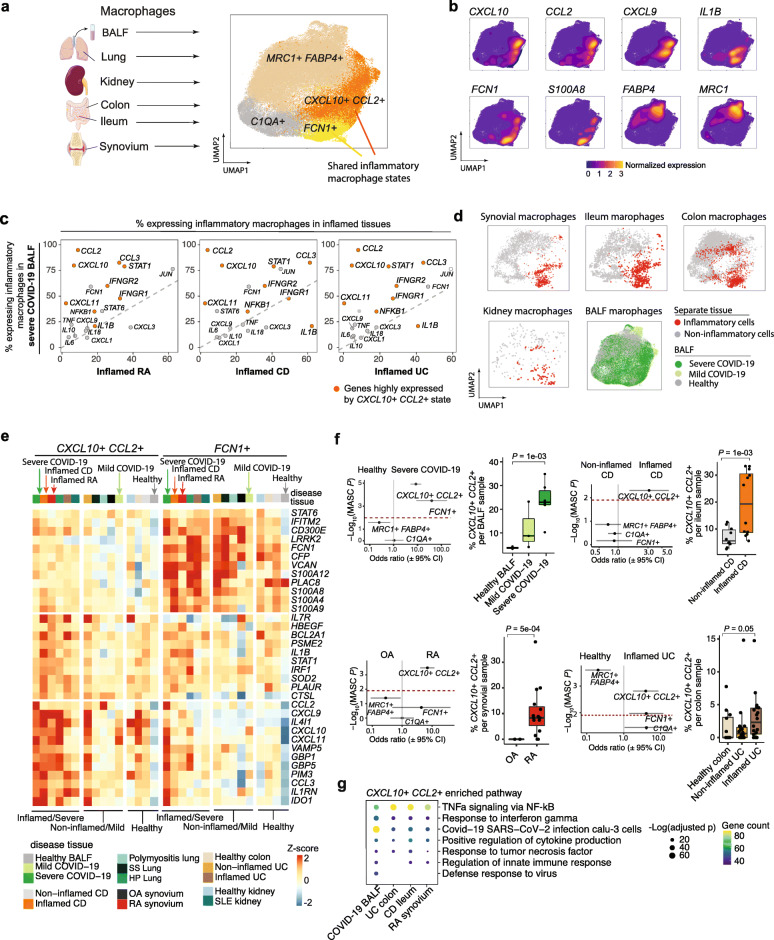
Integrative analysis of tissue-level macrophages reveals shared *CXCL10+ CCL2+* and *FCN1+* inflammatory macrophage states. **a** Integrative clustering of 74,373 macrophages from individuals from BALF, lung, kidney, colon, ileum, and synovium. **b** Density plot of cells with non-zero expression of marker genes in UMAP. **c** Proportion of inflammatory macrophages that express cytokines and inflammatory genes in severe COVID-19 compared to those in inflamed RA, CD, and UC. Orange represents *CXCL10+ CCL2+ *state-specific genes. **d** Previously defined inflammatory macrophages from diseased tissues are clustered with the majority of the macrophages from severe COVID-19. **e** *Z*-score of the pseudo-bulk expression of marker genes (AUC > 0.6 and Bonferroni-adjusted *P* < 10^−5^) for the *CXCL10+ CCL2+* and *FCN1+* macrophages. Columns show pseudo-bulk expression. **f** The proportions of *CXCL10+ CCL2+* macrophages of total macrophages per donor sample are shown from healthy BALF (*n* = 3), mild (*n* = 3), and severe (*n* = 6) COVID-19, non-inflamed CD (*n* = 10) and inflamed CD (*n* = 12), OA (*n* = 2) and RA (*n* = 15), and healthy colon (*n* = 12), non-inflamed UC (*n* = 18), and inflamed UC (*n* = 18). Box plots summarize the median, interquartile, and 75% quantile range. *P* is calculated by Wilcoxon rank-sum test within each tissue. The association of each cluster with severe/inflamed compared to healthy control was tested. 95% CI for the odds ratio (OR) is given. MASC *P* is calculated using one-sided *F* tests conducted on nested models with MASC [36]. The clusters above the dashed line (Bonferroni correction) are statistically significant. Clusters that have fewer than 30 cells are removed. **g** GSEA analysis for each tissue revealed shared enriched pathways for *CXCL10+ CCL2+* macrophages: TNF-α signaling via NF-kB (Hallmark gene set), response to interferon gamma (GO:0034341), Covid-19 SARS-CoV-2 infection calu-3 cells (GSE147507 [39]), positive regulation of cytokine production (GO:0001819), response to tumor necrosis factor (GO:0034612), regulation of innate immune response (GO:0045088), and defense response to virus (GO: 0051607)

To resolve the heterogeneity within the macrophage compartment, we analyzed 74,373 macrophages from 108 donors and performed weighted PCA and fine clustering analysis to define shared and distinct states across diseases (Fig. 2a, Additional file 2: Figure S3a, Additional file 4: Table S3). We identified four shared macrophage states defined by different marker sets: (1) *CXCL10+ CCL2+* cells, (2) *FCN1+* cells, (3) *MRC1+ FABP4+* cells, and (4) *C1QA+* cells (Fig. 2a, b, Additional file 2: Figure S3b). The *CXCL10+ CCL2+* cells and the *FCN1+* cells expressed classic inflammatory genes [15] including *IL1B*, *S100A8*, *CCL3, CXCL11, STAT1, IFNGR1,* and *NFKB1* (Fig. 2b, c). A higher proportion of inflammatory macrophages in severe COVID-19 expressed these inflammation-associated genes compared to healthy BALF (Additional file 2: Figure S3c). We detected the gene signature for the *CXCL10+ CCL2+* inflammatory macrophage state in a higher proportion of macrophages from severe COVID-19 BALF than from other inflamed tissues (Fig. 2c).

Liao et al. [4] previously identified *CXCL10+ CCL2+* and *FCN1*+ populations as inflammatory states in the COVID-19 BALF samples used in this integrated analysis. In our multi-disease clustering, the inflammatory macrophages from inflamed RA synovium and UC and CD intestinal tissue largely mapped to the same two inflammatory macrophages seen in severe COVID-19 (Fig. 2d, Additional file 2: Figure S3d-e). In most tissue types, we found all four states represented in all six tissues, and we quantified this overlap with LISI and estimated the variance explained in the PC space (Additional file 2: Figure S3f, g). Strikingly, we observed that the *FCN1+* inflammatory macrophage state dominated in SLE kidney, with few in the *CXCL10+ CCL2+* macrophages (Fig. 2d), suggesting that our integrative analysis was effective in identifying both shared inflammatory states while maintaining distinct patterns in a subset of tissues.

To comprehensively define markers for the two inflammatory tissue macrophage states shared across COVID-19, RA, UC, and CD, we performed a pseudo-bulk differential expression analysis (“Methods,” Additional file 5: Table S4, fold change > 2, AUC > 0.6, Bonferroni-adjusted *P* < 10^−5^). The CX*CL10+ CCL2+* inflammatory macrophages displayed significantly higher expression of *CXCL10*, *CXCL11*, *CCL2*, *CCL3*, *GBP1*, and *IDO1* in severe COVID-19, inflamed RA, and CD compared to the *FCN1+* macrophages (Fig. 2e). In contrast, the *FCN1+* macrophages displayed high expression of *FCN1* (Ficolin-1) and a series of alarmins such as *S100A8* and *S100A9* in most of the inflamed tissues (Fig. 2e). Both inflammatory macrophage states showed high expression of transcription factors that promote a pro-inflammatory macrophage phenotype, *STAT1* and *IRF1*, in inflamed RA, UC, CD, and COVID-19 BALF relative to healthy or non-inflamed tissues (Fig. 2e). Within the *CXCL10+ CCL2+* state, there was notable heterogeneity across cells in terms of *IL1B* expression indicating the possibility of further delineation of this macrophage state (Additional file 2: Figure S4a-b). Moreover, the effect size of all genes in *CXCL10+ CCL2+* and *FCN1+* subsets compared with the *MRC1+ FABP4+* macrophages for each tissue further highlighted a similar set of inflammatory genes with greatest fold changes across all diseases for each subset (Additional file 2: Figure S5).

As validation, we assessed the macrophage phenotypes found in a recent analysis of single cells from severe COVID-19 BALF [29]. Notably, we observed a significant correlation between the cross-disease shared *CXCL10+ CCL2+* macrophages and two monocyte-derived alveolar macrophage (MoAM) inflammatory phenotypes from this independent severe COVID-19 cohort (wherein they were referred to as MoAM1 and MoAM2) [29] (Additional file 2: Figure S6a-d). We further examined *CXCL10+ CCL2+* macrophage-associated genes with CD14+ cells from inflamed (leukocyte-rich) RA, non-inflamed (leukocyte-poor) RA, and OA [13]; we observed significant enrichment of *CXCL10+ CCL2+* state-specific genes (*CXCL10*, *CXCL9*, *CCL3*, *GBP1*, and *IDO1*), *FCN1+* state-specific genes (*FCN1*, *S100A9*, *CD300E*, *IFITM3*, and *CFP*), and genes (*IRF1*, *BCL2A1*, and *STAT1*) associated with both states in the macrophages from inflamed RA compared to non-inflamed RA and OA (Additional file 2: Figure S6e). By integrating macrophages across multiple inflamed tissues, we show that inflammatory subsets identified in COVID-19 may share common phenotypes with macrophages from other inflammatory conditions.

To elucidate cell states that were phenotypically associated, we tested the association of each state with severe COVID-19 compared to healthy BALF using a logistic regression model accounting for technical cell-level and donor-specific effects [36] (“Methods”). We observed the *CXCL10+ CCL2+* and *FCN1+* states are abundant in severe COVID-19 compared to healthy BALF (Fig. 2f). The *CXCL10+ CCL2+* inflammatory state was also expanded in inflamed CD compared to non-inflamed CD, RA compared to non-inflammatory OA, and inflamed UC compared to healthy colon, respectively (Fig. 2f). We indeed observed significant enrichment of the TNF-alpha signaling via nuclear factor-κB (NF-kB) pathway and the response to interferon gamma pathway in the *CXCL10+ CCL2+* cells from examined inflamed tissues (Fig. 2g). Consistent with this result, we also observed reduced frequencies of *MRC1+ FABP4+* macrophages in each inflamed tissue (Fig. 2f). Taken together, these results indicate that the shared *CXCL10+ CCL2+* inflammatory macrophage phenotype is expanded in inflamed tissues and severe COVID-19 BALF.

### Tissue inflammatory conditions that drive distinct macrophage phenotypes

**Fig. 3:**
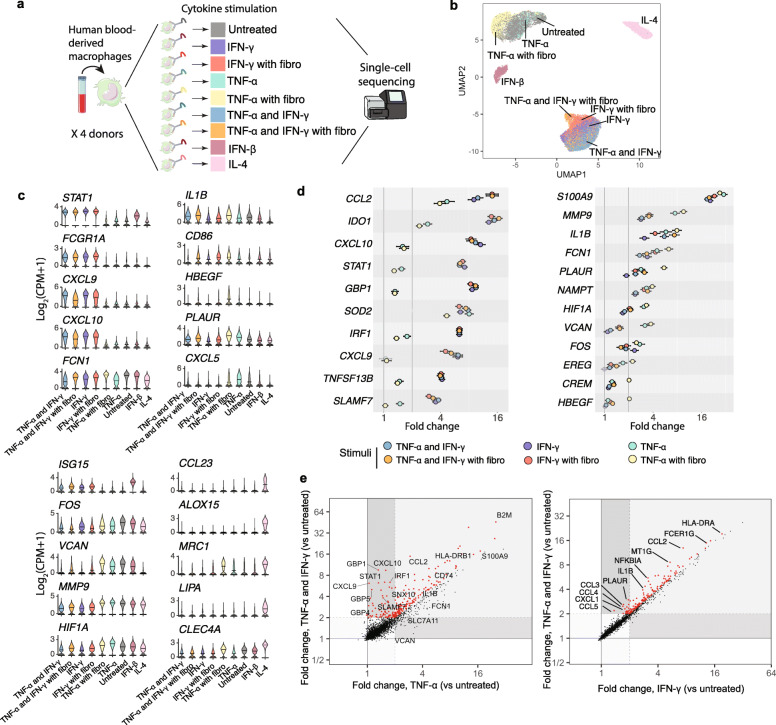
Human blood-derived macrophages stimulated by eight mixtures of inflammatory factors reveal heterogeneous macrophage phenotypes. **a** Schematic representation of the single-cell cell hashing experiment on human blood-derived macrophages stimulated by eight mixtures of inflammatory factors from 4 donors. A single-cell antibody-based hashing strategy was used to multiplex samples from different stimulatory conditions in one sequencing run. Here fibro denotes fibroblasts. **b** The 25,823 stimulated blood-derived macrophages from 4 donors are colored and labeled in UMAP space. **c** Log-normalized expression of genes that are specific to different conditions are displayed in violin plots. Mean of normalized gene expression is marked by a line and each condition by individual coloring. CPM denotes counts per million. **d** Stimulation effect estimates of genes that are most responsive to conditions with IFN-γ or TNF-α with fibroblasts comparing to untreated macrophages are obtained using linear modeling. Fold changes with 95% CI are shown. **e** Fold changes in gene expression after TNF-α and IFN-γ stimulation vs. TNF-α stimulation (left), and TNF-α and IFN-γ vs. IFN-γ stimulation (right) for each gene. Genes in red have fold change > 2, Bonferroni-adjusted *P* < 10^−7^, and a ratio of TNF-α and IFN-γ fold change to TNF-α fold change greater than 1 (left) or a ratio of TNF-α and IFN-γ fold change to IFN-γ fold change greater than 1 (right). Genes that are most responsive to either IFN-γ (left) or TNF-α (right) are labeled

To define the factors that shape disease-associated macrophage states in affected tissues, we generated human blood-derived macrophages from four donors and activated them with eight defined mixtures of inflammatory factors, focusing particularly on the effects of antiviral interferons (IFN-β and IFN-γ) and pro-inflammatory cytokines such as TNF that mediate CRS and tissue pathology in RA and IBD [40] (Fig. 3a, Additional file 2: Figure S7a, “Methods”). Co-cultured fibroblasts were a component in some conditions to generate factors produced by resident stroma. To reduce confounding batch effects during scRNA-seq barcode labeling, we used a single-cell antibody-based hashing strategy [41] to multiplex samples from different stimulatory conditions in one sequencing run (Additional file 6: Table S5, Additional file 7: Table S6). We obtained 25,823 post-QC cells after applying 10X Genomics droplet-based single-cell assay (Additional file 2: Figure S7b-d, “Methods”). In the UMAP space, a strong response to IFN-γ drove much of the observed variation; cells treated with IFN-γ clustered well apart from all other conditions (Fig. 3b). All conditions containing IFN-γ (Type II interferon) resulted in macrophages with high expression levels of the transcription factor *STAT1*, interferon-stimulated genes *CXCL9* and *CXCL10,* and inflammatory receptors such as *FCGR1A* [42] (Fig. 3c). Consistent with well-established effects, macrophages stimulated by TNF induced *MMP9*, *IL1B,* and *PLAUR* expression while IL-4 stimulation increased expression of *CCL23*, *MRC1*, and *LIPA* (Fig. 3c).

Using linear models, we identified the genes with the greatest changes in expression after each stimulation and estimated the effect sizes (“Methods”). We found that 403 genes (fold change > 2, FDR < 0.05) were significantly enriched in the TNF-α and IFN-γ stimulation compared to untreated macrophages. All conditions with IFN-γ resulted in similar effect sizes for induction of *CCL2*, *CXCL9*, *CXCL10*, *SLAMF7*, and *STAT1* expression—indicating a robust IFN-γ driven macrophage signature (Fig. 3d left, Additional file 2: Figure S7e). This included robust induction by IFN-γ in macrophages co-treated with TNF (Fig. 3e left). Collectively, the TNF-driven gene expression patterns appeared more modifiable by co-stimulatory factors than IFN-γ. For example, co-cultured fibroblasts further increased TNF-induced *MMP9*, *PLAUR,* and *VCAN* expression, while co-stimulating with IFN-γ repressed TNF induction of these genes (Fig. 3d right). Nonetheless, a portion of the TNF effect was well preserved in TNF plus IFN-γ co-stimulated cells, including genes such as *CCL2*, *CCL3*, *IL1B*, and *NFKBIA* (Fig. 3e right). TNF-α and IFN-γ ultimately generated a macrophage phenotype with increased expression of NF-kB targets such as *NFKBIA*, *IL1B*, and *HLA-DRA* together with STAT1 targets such as *CXCL9* and *CXCL10,* and *GBP1* and *GBP5* (Fig. 3d, e)*.*

### Identification of an IFN-γ and TNF-α synergistically driven inflammatory macrophage phenotype expanded in severe COVID-19 lungs and other inflamed disease tissues

**Fig. 4:**
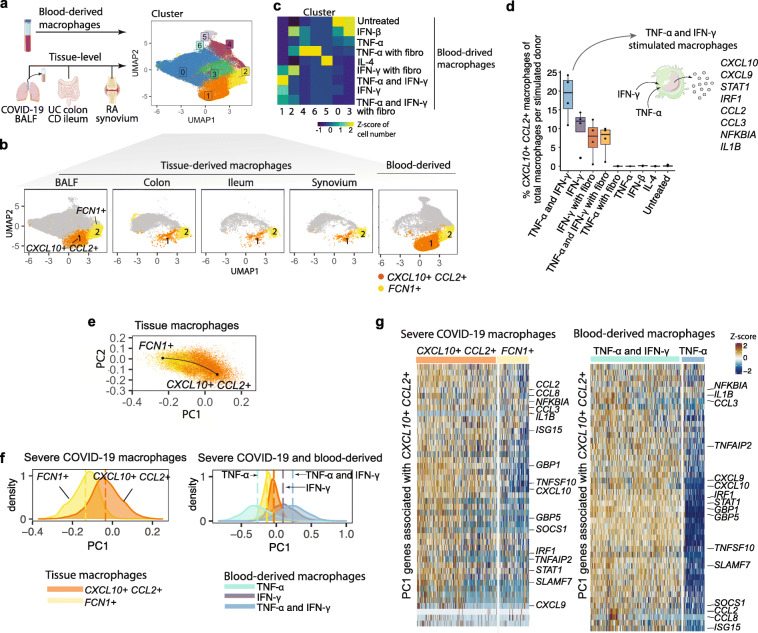
TNF-α and IFN-γ driven *CXCL10+ CCL2+* macrophages are expanded in severe COVID-19 and other inflamed tissues. **a** Integrative clustering of stimulated blood-derived macrophages with tissue-level macrophages from COVID-19 BALF, UC colon, CD ileum, and RA synovium. **b** The previously identified tissue-level *CXCL10+ CCL2+* state corresponds to cluster 1 (orange), and the *FCN1+* inflammatory macrophage state corresponds to cluster 2 (yellow). Macrophages from each tissue source are displayed separately in the same UMAP coordinates as in **a**. **c** Heatmap indicates the concordance between stimulatory conditions and integrative cluster assignments. *Z*-score of the number of cells from each stimulatory condition to the integrative clusters is shown. **d** For the blood-derived stimulated macrophages, the proportions of *CXCL10+ CCL2+* macrophages of total macrophages per stimulated donor are shown. **e** PCA analysis on the identified inflammatory macrophages. The first PC captures a gradient from the *FCN1+* state to the *CXCL10+ CCL2+* state. **f** Upon this, macrophages from severe COVID-19 mapped to PC1 present a shift in cell frequency between the *FCN1+* and *CXCL10+ CCL2+* (Wilcoxon rank-sum test *P* = 1.4e−07). The TNF-α stimulated macrophages (mean − 0.27) were projected to the left of the *FCN1+* tissue macrophages (mean − 0.14), while the IFN-γ (mean 0.10), and TNF-α and IFN-γ (mean 0.23), stimulated macrophages were projected to the right of the *CXCL10+ CCL2+* tissue macrophages (− 0.03). **g** Genes associated with *CXCL10+ CCL2+* driven by PC1 show high expression levels on the severe COVID-19 macrophages and also TNF-α and IFN-γ stimulated blood-derived macrophages. We recapitulate the gradient observed in vivo across multiple diseases by stimulating macrophages ex vivo with synergistic combinations of TNF-α and IFN-γ

Our cross-tissue integrative analysis revealed two shared inflammatory macrophage states (Fig. 2). To further understand these cell states and the in vivo inflammatory tissue factors driving them, we integrated the single-cell transcriptomes of both the tissue macrophages and our experimentally stimulated macrophages. After combining and correcting for tissue, technology, and donor effects, we identified 7 distinct macrophage clusters (Fig. 4a). We evaluated the robustness of the clustering and observed that our clusters were stable to the choice of the variable genes used in the analysis (Additional file 2: Figure S8a). The tissue *CXCL10+ CCL2+* inflammatory macrophages from UC colon, CD ileum, RA synovium, and COVID-19 BALF were transcriptionally most similar to macrophages stimulated by the combination of TNF-α plus IFN-γ in cluster 1 (Fig. 4b, c, Additional file 2: Figure S8b-c). The blood-derived macrophages in cluster 1 included macrophages stimulated by four different conditions all including IFN-γ, of which the most abundant population (37.5%) were macrophages stimulated by TNF-α with IFN-γ (Fig. 4c, d). Comparing our results to a previously reported macrophage spectrum with 28 unique stimulatory conditions [11], we observed the highest expression of cluster 1-associated genes in their macrophages exposed to conditions including both TNF and IFN-γ (Additional file 2: Figure S9a).

We further identified a principal component (PC1) that captures a gradient from the *FCN1+* state to the *CXCL10+ CCL2+* state by applying PCA analysis to the tissue-level inflammatory macrophages (Fig. 4e), suggesting a potential continuum between the inflammatory *FCN1+* and *CXCL10+ CCL2+* states. Aligning cells from separate tissues along PC1, we found that the majority of inflammatory macrophages in RA, UC, and CD align more closely with the *FCN1+* state (Additional file 2: Figure S9b). In severe COVID-19, we observed a shift in cell frequency between the *FCN1+* and *CXCL10+ CCL2+* macrophages (Wilcoxon rank-sum test *P* = 1.4e−07, Fig. 4f). Furthermore, we mapped the experimentally stimulated blood-derived macrophages to PC1 based on the top 50 genes with the largest and smallest PC1 gene loadings. Strikingly, the TNF-α stimulated macrophages (mean − 0.27) map to the left of the *FCN1+* tissue macrophages (mean − 0.14), while the IFN-γ (mean 0.10), and TNF-α and IFN-γ (mean 0.23), stimulated macrophages map to the right of the *CXCL10+ CCL2+* tissue macrophages (− 0.03) (Fig. 4f). This suggests the importance of IFN-γ stimulation in order to drive a phenotype most similar to the *CXCL10+ CCL2+* state, with the addition of TNF stimulation resulting in further pushing of the macrophage phenotype along the PC1 trajectory. We observed higher expression levels of PC1-associated genes, for example *CXCL10*, *STAT1*, *CCL2*, *CCL3*, *NFKBIA*, and *GBP1*, in *CXCL10+ CCL2+* severe COVID-19 compared to *FCN1+* cells, and higher induced expression levels of these same genes in TNF-α and IFN-γ stimulation compared to TNF-α stimulation alone (Fig. 4g). Taken together, these results suggest we are able to recapitulate the gradient observed in vivo across multiple diseases by stimulating macrophages ex vivo with synergistic combinations of IFN-γ and TNF-α.

## Discussion

Our study demonstrates the power of a multi-disease reference dataset to interpret cellular phenotypes and tissue states, while placing them into a broader context that may provide insights into disease etiology and rationale for repurposing medications. Such meta-datasets can increase the resolution of cell states and aid understanding of shared cellular states found in less well-understood diseases such as COVID-19. Amassing diverse tissues from > 120 donors with a wide range of diseases, we built a human tissue inflammation single-cell reference. Applying powerful computational strategies, we integrated > 300,000 single-cell transcriptomes and corrected for factors that interfere with resolving cell-intrinsic expression patterns. In particular, we have identified a *CXCL10+ CCL2+* inflammatory macrophage phenotype shared between tissues affected in autoimmune disease (RA), inflammatory diseases (CD and UC), and infectious disease (COVID-19). We observed that the abundance of this population is associated with inflammation and disease severity. With integrated analysis of an ex vivo dataset, we elucidated its potential cytokine drivers: IFN-γ together with TNF-α.

Macrophages are ideal biologic indicators for the in vivo state of a tissue due to their dynamic nature, robust responses to local factors, and widespread presence in most tissues. Through our cross-disease analysis, we defined two inflammatory macrophage states that can be found in selected groups of seemingly unrelated tissues and diseases. Most notably, the *CXCL10+ CCL2+* inflammatory macrophages predominate in the bronchoalveolar lavage of patients with severe COVID-19, and are also detected in synovial tissue affected by RA and inflamed intestine from patients with IBD. These cells are distinguished by high levels of *CXCL10* and *CXCL11*, *STAT1*, *IFNGR1,* and *IFNGR2,* as well as *CCL2* and *CCL3*, *NFKB1*, *TGFB1*, and *IL1B*. This gene expression pattern of the JAK/STAT and NF-kB-dependent cytokines implicates induction by an intriguing combination of both the IFN-induced JAK/STAT and TNF-induced NF-kB pathways and, in conjunction, the overall transcriptome program most closely aligns with macrophages stimulated by IFN-γ plus TNF-α. As both JAK inhibitors and anti-TNF medications have outstanding efficacy in treating RA and anti-TNFs are the most common medications treating inflammatory bowel disease, including Crohn’s Disease [2], these therapies may target the inflammatory macrophages in severe COVID-19 lung during the phase involving cytokine release syndrome [43].

Infection with SARS-CoV2 triggers local immune response and inflammation in the lung compartment, recruiting macrophages that release and respond to inflammatory cytokines and chemokines [6]. This response may change with disease progression, in particular during the transition towards the cytokine storm associated with severe disease. Intriguingly, our cross-disease tissue study strongly suggests that IFN-γ is an essential component in the inflammatory macrophage phenotype in severe COVID-19. Most studies on interferons and coronaviruses have focused on Type I interferons, such as IFN-β, due to their robust capacity to interfere with viral replication [44]. Indeed, ongoing research into the administration of recombinant IFN-β has shown promise in reducing the risk of severe COVID-19 disease [45]. However, other studies have indicated that targeting IFN-γ may be an effective treatment for cytokine storm, a driver of severe disease in COVID-19 patients [46, 47]. Additionally, several studies have indicated that targeting IFN-γ using JAK inhibitors such as ruxolitinib, baricitinib, and tofacitinib offers effective therapeutic effects in treating severe COVID-19 patients [43, 48–51]. Clinical trials of Type II interferon inhibitors in COVID-19 are under way (NCT04337359, NCT04359290, and NCT04348695) [43]. Recent research has also identified that the synergism of TNF-α and IFN-γ can trigger inflammatory cell death, tissue damage, and mortality in SARS-CoV-2 infection [52], and shown increased levels of IFN-γ, TNF-α, CXCL10, and CCL2 in the serum of severe COVID-19 patients [53]. In agreement with these studies, our findings indicate that IFN-γ is an important mediator together with TNF-α of severe disease, in part through activating the inflammatory *CXCL10+ CCL2+* macrophage subset. We hypothesize that anti-Type II interferon (like JAK inhibitors) and anti-TNF combinatorial treatment might prove effective at inhibiting the cytokine storm driving acute respiratory distress syndrome in patients with severe COVID-19. We are aware of the limited number of longitudinal BALFs from COVID-19 patients involved in our across-tissue study due to the current crisis situation, so we expect to replicate our findings in a broader generalization of COVID-19 patients in the future. Of course, the presence of an IFN-γ and TNF phenotype is an association that may not be causal. Whether targeting these cytokines is reasonable or not will depend on additional clinical investigation.

## Conclusions

In this study, we built a single-cell immune reference from multiple inflamed disease tissues and identified two inflammatory macrophage states, *CXCL10+ CCL2+* and *FCN1+* inflammatory macrophages, that were shared between COVID-19 and inflammatory diseases such as RA, CD, and UC. We demonstrated that the *CXCL10+ CCL2+* macrophages are transcriptionally similar to human blood-derived macrophages stimulated by IFN-γ and TNF-α and were expanded in severe COVID-19 lungs and inflamed RA, CD, and UC tissues. This finding indicates that Type II interferon and TNF responses may be involved in late-stage cytokine storm-driven severe COVID-19 and inhibiting these responses in the inflammatory macrophages may be a promising treatment. Our cross-tissue single-cell integrative strategy along with our disease association analysis provides a proof-of-principle that identifying shared pathogenic features across human inflamed tissues and COVID-19 lungs has the potential to guide drug repurposing.

## Supplementary Information

**Additional file 1: Table S1.** Basic information and demography of multiple single-cell datasets.

**Additional file 2: Figure S1.** Overall integration of immune cells from multiple scRNA-seq datasets. **Figure S2.** Quantification of the performance of all cell type multi-disease tissue integration. **Figure S3.** Tissue-level macrophage integrative analysis of multiple scRNA-seq datasets. **Figure S4.** Heterogeneity of shared inflammatory macrophages from multiple tissues. **Figure S5.** Single-cell differential gene expression analysis of comparing inflammatory macrophages with non-inflammatory macrophages within each individual tissue source. **Figure S6.** Examination of the *CXCL10+ CCL2+* macrophage marker genes in additional diseased cohort studies. **Figure S7.** Experimental design and quality control of human blood-derived macrophages stimulated by different conditions. **Figure S8.** Integrative analysis of tissue-level macrophages and human blood-derived macrophages. **Figure S9.** Assessment of previously reported stimulated macrophage spectrum analysis and alignment of macrophages from different disease tissues to a trajectory.

**Additional file 3: Table S2.** Cell type marker genes and statistics.

**Additional file 4: Table S3.** Number of cells per cluster, per disease and tissue for macrophage integration analysis.

**Additional file 5: Table S4.** Macrophage cluster marker genes and relative statistics.

**Additional file 6: Table S5.** Hashtag antibodies for the 10X single-cell cell hashing experiment.

**Additional file 7: Table S6.** Details for the 10X single-cell cell hashing experiment.

## Data Availability

The single-cell RNA-seq data for blood-derived macrophages are available in the Gene Expression Omnibus database with accession number GSE168710, https://www.ncbi.nlm.nih.gov/geo/query/acc.cgi?acc=GSE168710 [54]. Source code repository to reproduce analyses is located at https://github.com/immunogenomics/inflamedtissue_covid19_reference [55]. The publicly available datasets analyzed during the study are available from the GEO repository: GSE134809 (https://www.ncbi.nlm.nih.gov/geo/query/acc.cgi?acc=GSE134809) [27] GSE122960 (https://www.ncbi.nlm.nih.gov/geo/query/acc.cgi?acc=GSE122960) [28] GSE145926 (https://www.ncbi.nlm.nih.gov/geo/query/acc.cgi?acc=GSE145926) [4] GSE155249 (https://www.ncbi.nlm.nih.gov/geo/query/acc.cgi?acc=GSE155249) [29] GSE47189 (https://www.ncbi.nlm.nih.gov/geo/query/acc.cgi?acc=GSE47189) [11] dbGap repository: phs001457.v1.p1 (https://www.ncbi.nlm.nih.gov/projects/gap/cgi-bin/study.cgi?study_id=phs001457.v1.p1) [13] phs001529.v1.p1 (https://www.ncbi.nlm.nih.gov/projects/gap/cgi-bin/study.cgi?study_id=phs001529.v1.p1) [25] phs001457.v1.p1 (https://www.ncbi.nlm.nih.gov/projects/gap/cgi-bin/study.cgi?study_id=phs001457.v1.p1) [26] Single Cell Portal: SCP259 (https://singlecell.broadinstitute.org/single_cell/study/SCP259/intra-and-inter-cellular-rewiring-of-the-human-colon-during-ulcerative-colitis) [15]
