# Supplementary material for: IFN-γ and TNF-α drive a CXCL10+ CCL2+ macrophage phenotype expanded in severe COVID-19 lungs and inflammatory diseases with tissue inflammation
Source: Genome Med. 2021 Apr 20;13:64. doi: 10.1186/s13073-021-00881-3 (PMC8057009; doi:10.1186/s13073-021-00881-3)
Supplement: Supplementary file 2 — Additional file 2: Figure S1. Overall integration of immune cells from multiple scRNA-seq datasets. Figure S2. Quantification of the performance of all cell type multi-disease tissue integration. Figure S3. Tissue-level macrophage integrative analysis of multiple scRNA-seq datasets. Figure S4. Heterogeneity of shared inflammatory macrophages from multiple tissues. Figure S5. Single-cell differential gene expression analysis of comparing inflammatory macrophages with non-inflammatory macrophages within each individual tissue source. Figure S6. Examination of the CXCL10+ CCL2+ macrophage marker genes in additional diseased cohort studies. Figure S7. Experimental design and quality control of human blood-derived macrophages stimulated by different conditions. Figure S8. Integrative analysis of tissue-level macrophages and human blood-derived macrophages. Figure S9. Assessment of previously reported stimulated macrophage spectrum analysis and alignment of macrophages from different disease tissues to a trajectory. [file 13073_2021_881_MOESM2_ESM.pdf]

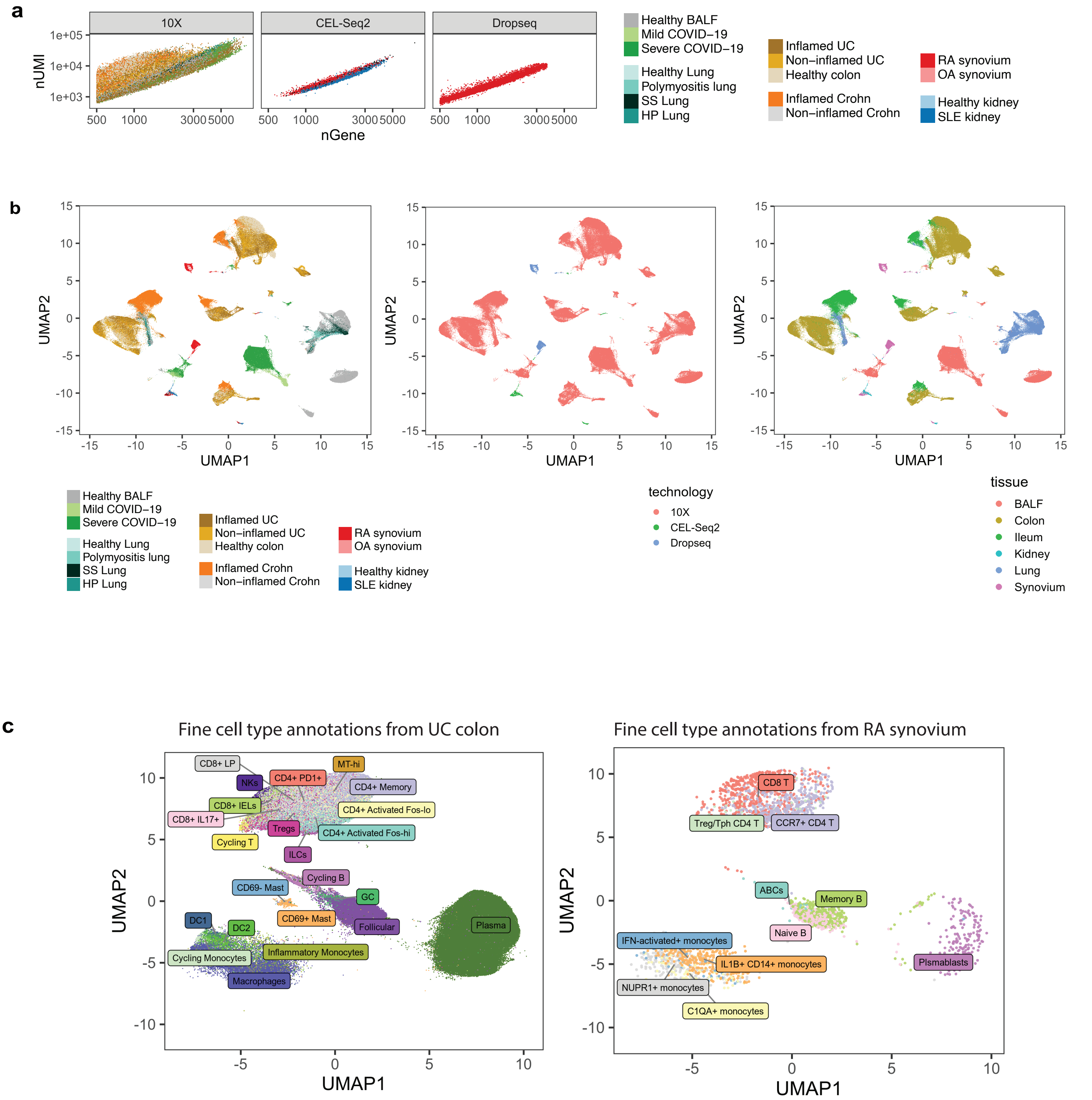

Fig S1. Overall integration of immune cells from multiple scRNA-seq datasets.
a. We describe the diversity of the single-cell sequencing technologies using nGene (number of genes detected) and nUMI (number of unique molecular identifiers). **b**. We show the disease category, sequencing technology, and tissue source for each cell in the UMAP space before correcting batch effects. Most of the cells are clustered by separate tissue sources rather than shared cell type populations before batch correction. **c**. We are also able to reconstruct the high-resolution of the immune cell subsets in the overall integrative embeddings. We color each cell by the original published cell subset annotations from UC colon and RA synovium.

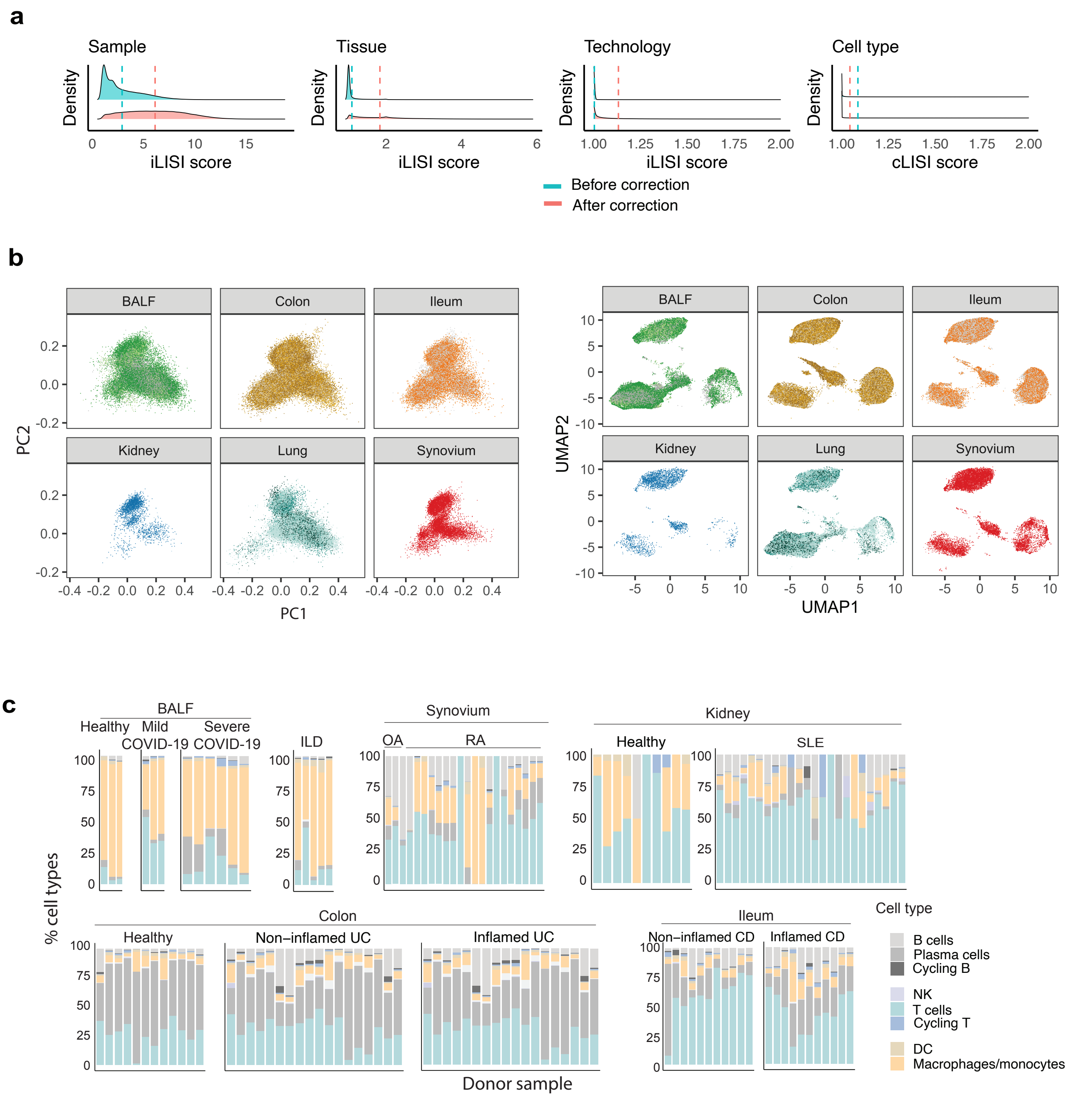

Fig S2. Quantification of the performance of all cell type multi-disease tissue integration.
a. We quantified the mixture level of donor samples and tissue sources using LISI score for before and after harmony batch effect correction. A iLISI (integration LISI) score of 1.0 denotes no mixing while in contrast, higher scores indicate better mixing of batches. We observed that applying Harmony increased mixing among donors (iLISI increasing from mean 2.9 to 6.1), tissue sources (iLISI increasing from mean 1.0 to 1.8), and technology (iLISI increasing from mean 1.0 to 1.5). A cLISI (cell type LISI) measures integration accuracy of pre-defined cell-type annotations. The cLISI score after batch correction decreased, which reflects a more accurate biological relevant cell type prediction after batch correction. **b**. After batch effect correction, we colored the cells from different disease tissue sources in the PCA and UMAP space. The same cell types from different tissue sources cluster together. **c**. Proportion of cell types based on cell frequency for each sample across all examined tissues.

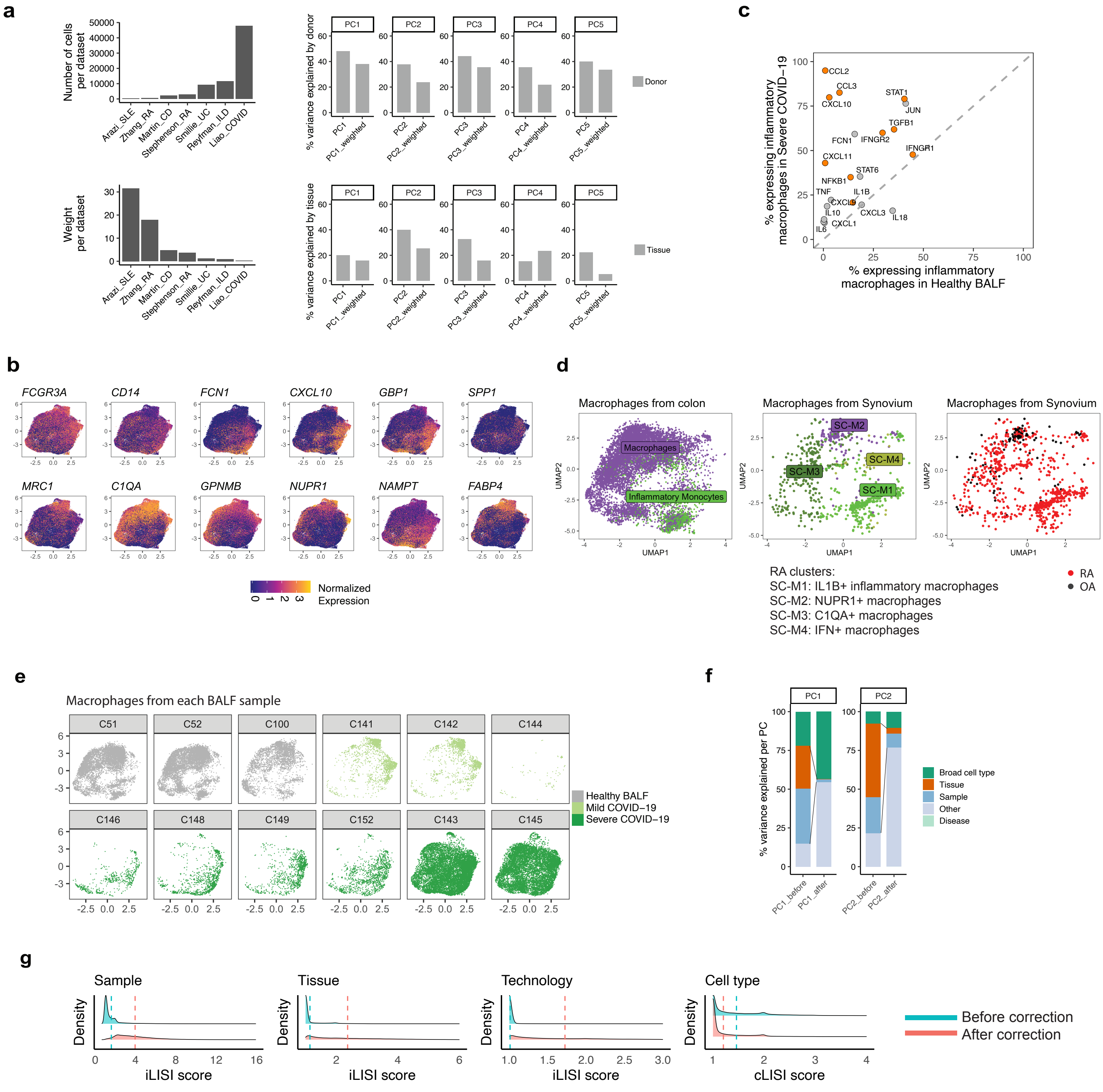

Fig S3. Tissue-level macrophage integrative analysis of multiple scRNA-seq datasets.
a. We quantified the percent of variance explained for top 5 PCs by weighted PCA vs standard PCA using R package limma. Smaller percentages of variance come from donor and tissue using weighted PCA compared to standard PCA. **b**. We show gene expression of macrophage cluster marker genes in the UMAP space. **c**. Proportion of expressing (non-zero) inflammatory cytokines and genes from inflammatory macrophages in severe COVID-19 are higher compared to healthy BALF. Genes that are highly expressed in the *CXCL10+ CCL2+* inflammatory macrophages are highlighted in orange. **d**. Original macrophage subset annotations are projected into the integrative UMAP embeddings. The previously identified inflammatory macrophages from UC colon and RA synovium are colored and labeled. Cells from RA (red) and OA (black) synovium are colored. **e**. Individual donors from healthy, mild and severe COVID-19 BALF are shown separately based on the same integrative UMAP coordinates. **f**. Proportion of variance explained by pre-defined macrophage subset, tissue, sample, and technology for PC1 and PC2 in the gene expression space before and after batch effect correction. **g**. We use iLISI score to quantify the mixture level of sample, tissue, technology, and cLISI score to measure the integration accuracy of pre-define macrophage subsets for before and after batch correction.

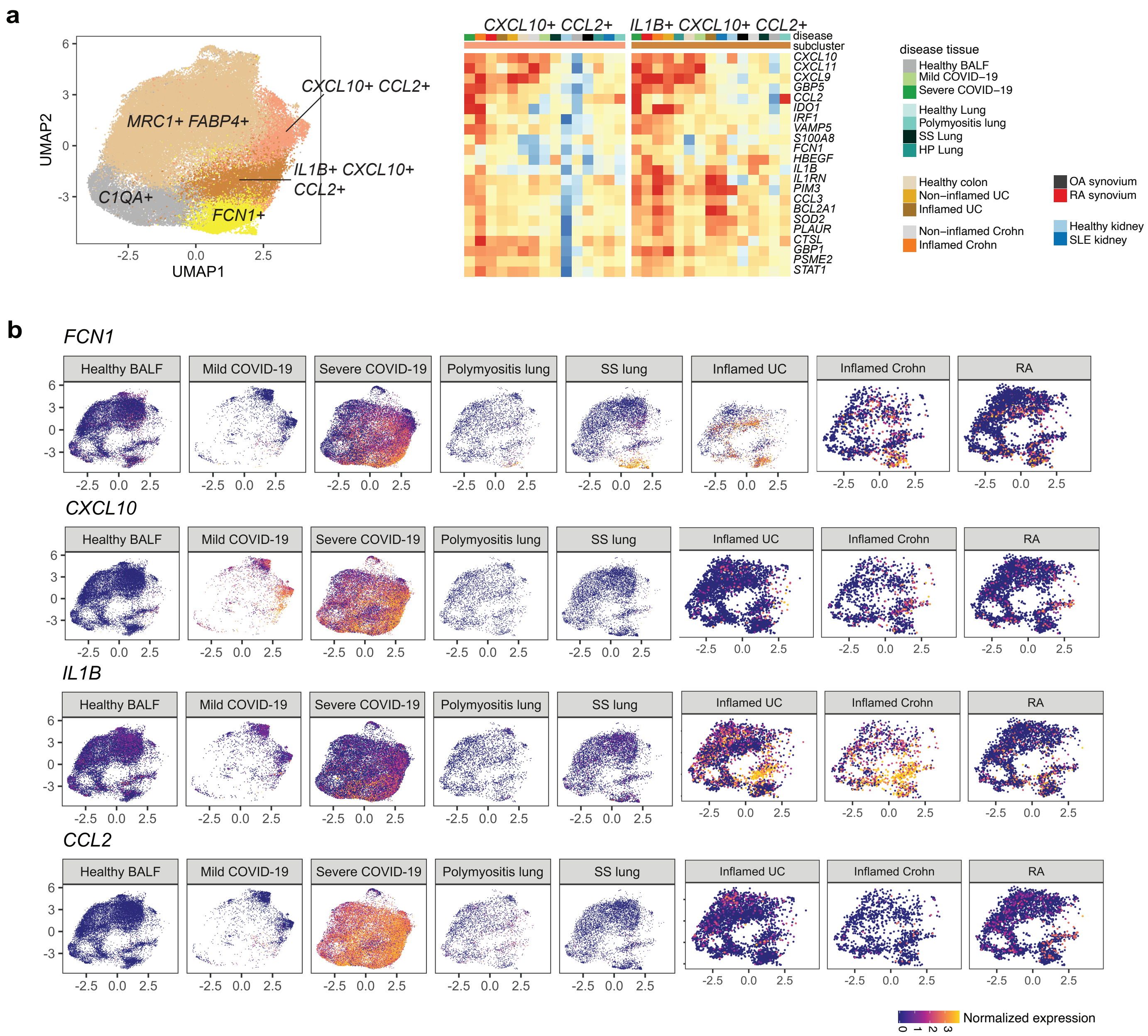

Fig S4. Heterogeneity of shared inflammatory macrophages from multiple tissues.
a. Heterogeneity of the identified *CXCL10+ CCL2+* inflammatory macrophages correlates with IL1B expression.
b. We show the expression of *FCN1*, *CXCL10*, *IL1B*, and *CCL2* for different disease tissue sources.

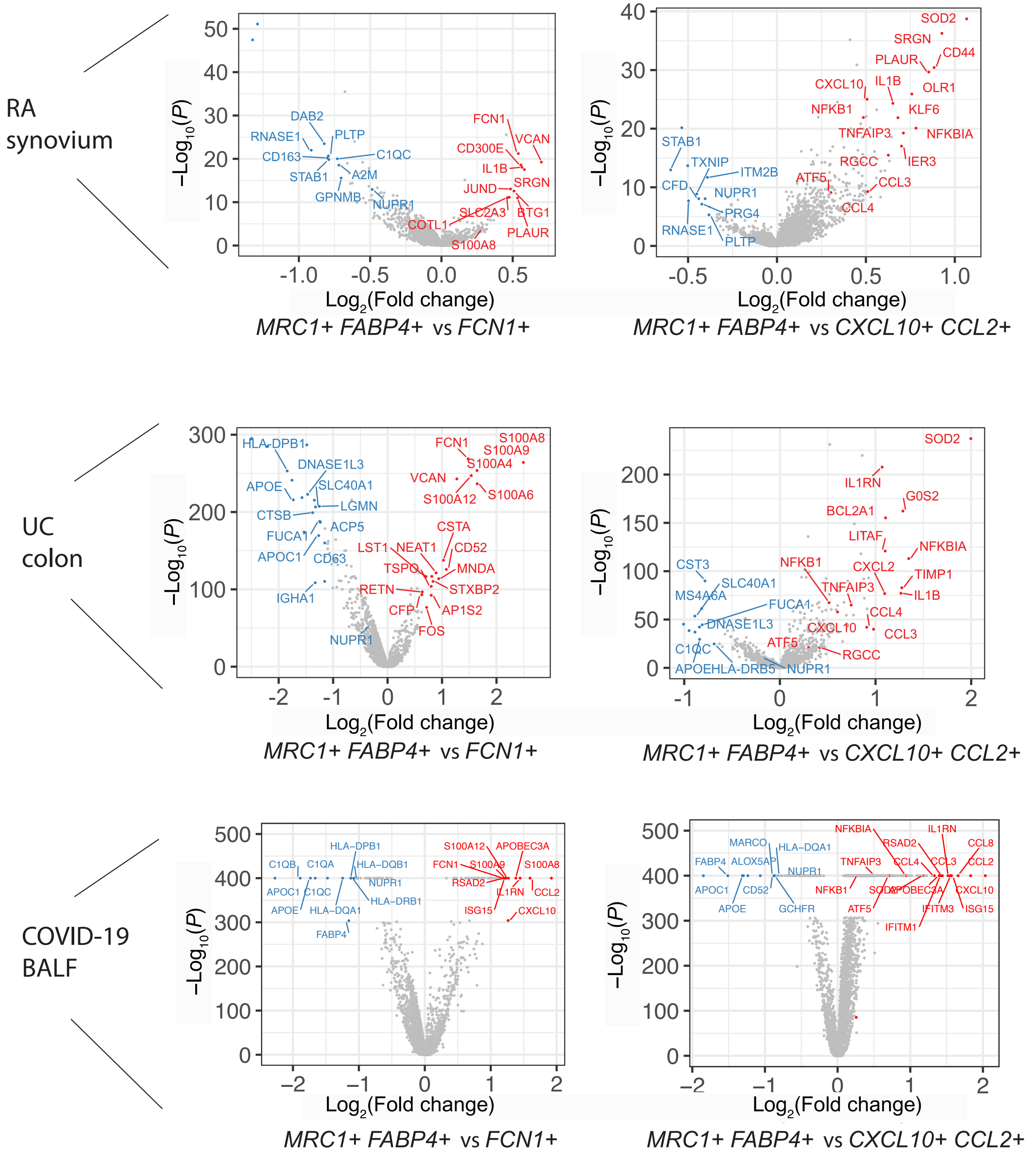

Fig S5. Single-cell differential gene expression analysis of comparing inflammatory macrophages with non-inflammatory macrophages within each individual tissue source.
Log-transformed fold change and P value for each gene are shown in volcano plots. Within RA synovium, we presented the differential gene expression analysis by comparing *FCN1*+ macrophages with *MRC1*+ *FABP4*+ macrophages from the same RA synovium, and also *CXCL10*+ *CCL2*+ macrophages with *MRC1*+ *FABP4*+ macrophages from the same RA synovium. We performed similar differential gene expression analysis for UC colon and COVID-19 BALF.

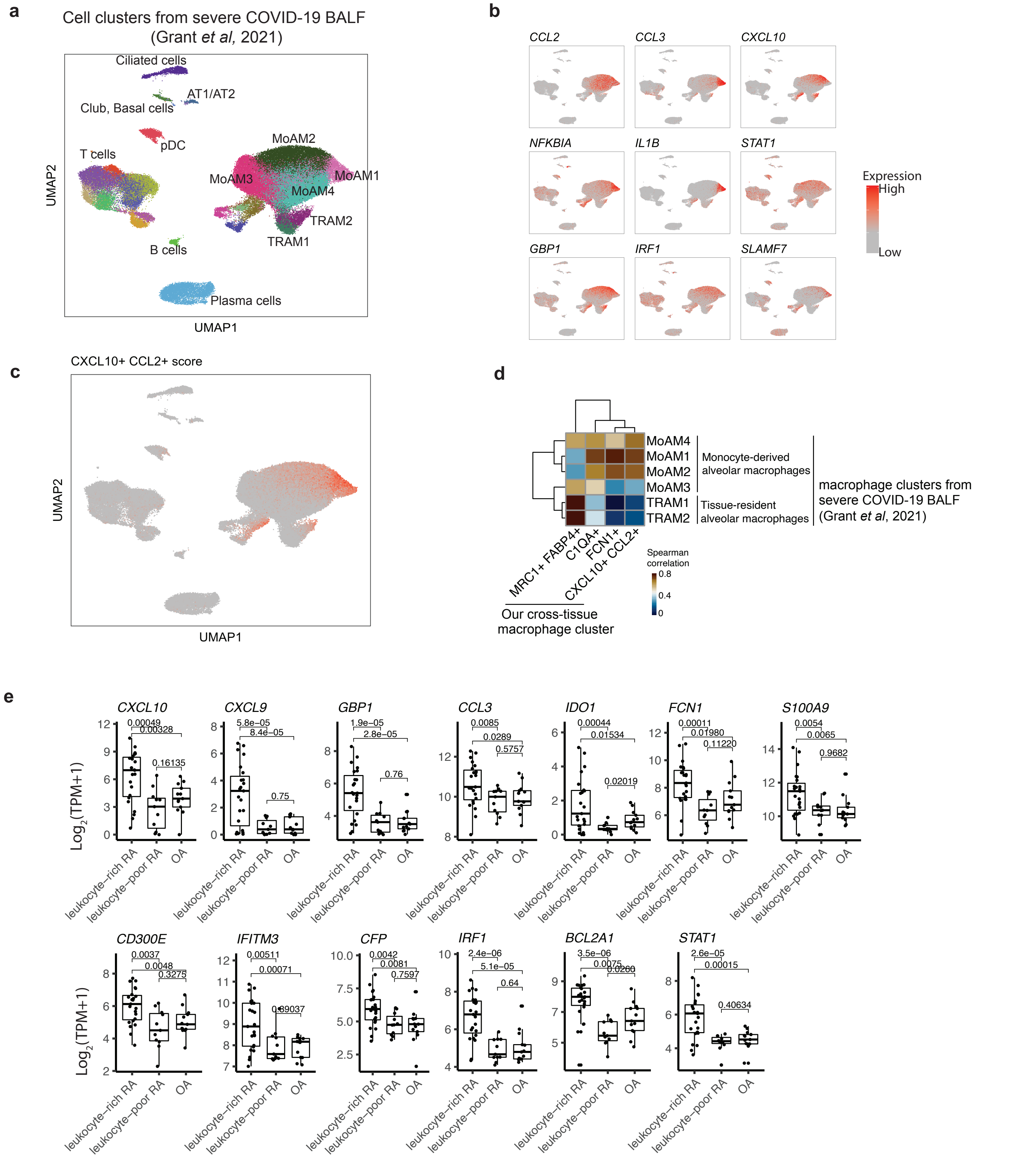

Fig S6. Examination of the *CXCL10+ CCL2+ macro*phage marker genes in additional diseased cohort studies.
a-c. We show the expression of *CXCL10+ CCL2+* marker genes and create a *CXCL10+ CCL2+* gene score for each cell from monocyte-derived alveolar macrophages (MoAM1, MoAM2, MoAM3, and MoAM4) and tissue-resident macrophages (TRAM1 and TRAM2) from severe COVID-19 BALF (GSE155249, Grant, *et al*, 2021). **d.** We observed a significant correlation between our *CXCL10+ CCL2+* macrophages with the monocyte-derived alveolar macrophages (MoAM1 and MoAM2) from Grant, *et al*, 2021. **e.** Bulk RNA-seq gene expression of inflammatory macrophage-associated genes using CD45+ CD14+ flow sorted macrophages from RA and OA synovium (ImmPort SDY998). We show the expression of genes that are specific to the *CXCL10+ CCL2+* and *FCN1+* from each RNA-seq sample in the leukocyte-rich (n=23), leukocyte-poor (n=11), and OA (n=13) groups. TPM: transcript count per million. Dots represent samples, lines represent means. Wilcoxon rank-sum test is applied.

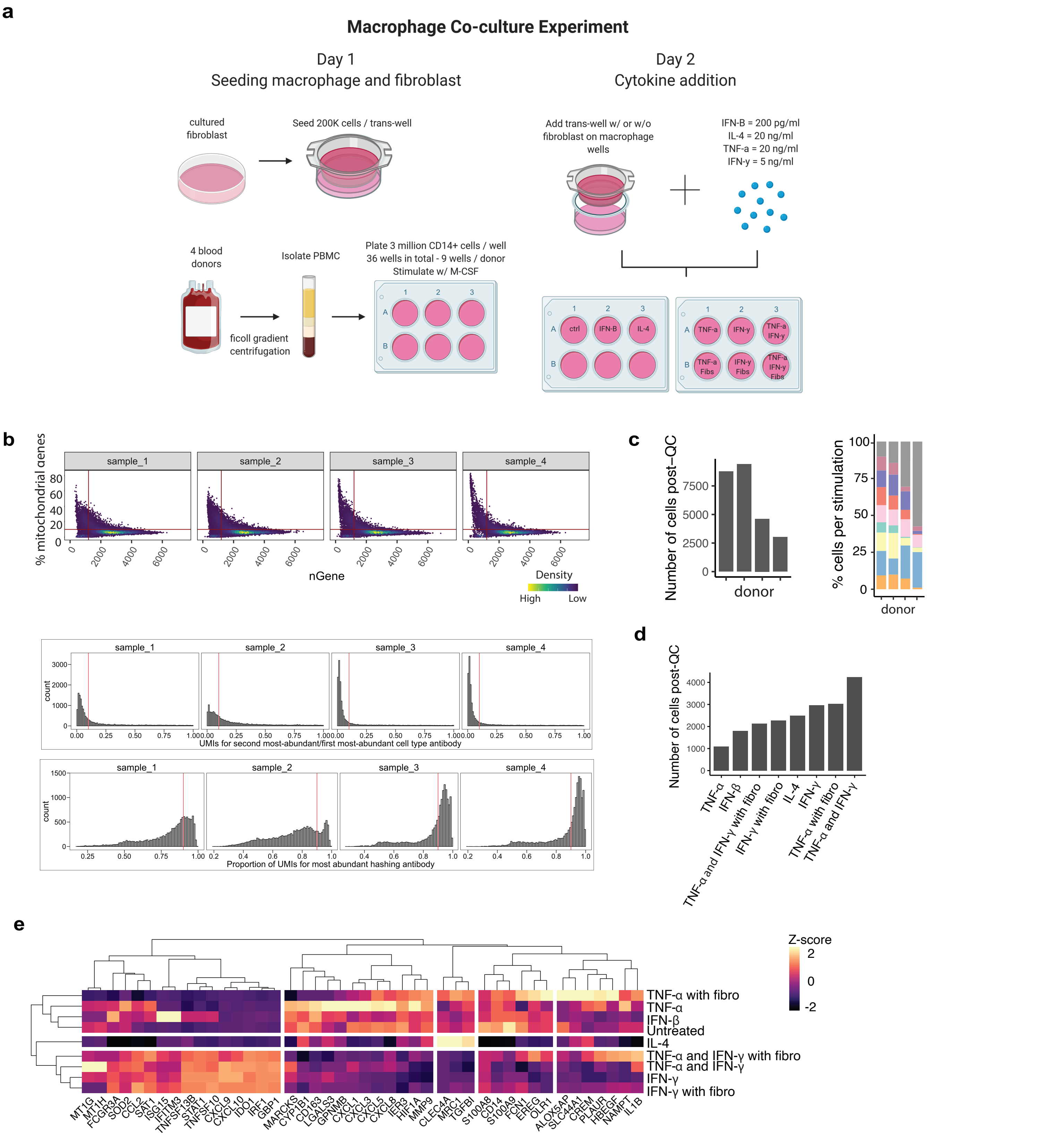

Fig S7. Experimental design and quality control of human blood-derived macrophages stimulated by different conditions. **a**. Schematic plot of experimental design for the human blood-derived macrophages. Seeding macrophages and fibroblasts, addition of cytokines, and single-cell hashing tag steps were included for the experiment. **b**. We filtered cells expressing fewer than 1,000 nGene or with more than 10% of UMIs mapping to mitochondrial genes. We also filtered cells whose proportion of UMIs for the most abundant hashing antibody was less than 90%; and removed cells whose ratio of the second most-abundant to first most-abundant antibody was greater than 10%. **c**. Number of cells and proportion of different stimulatory conditions for each donor. **d**. Number of cells from each stimulatory condition. **e**. Heatmap illustrating Z-score of the normalized gene expression for condition-specific genes.

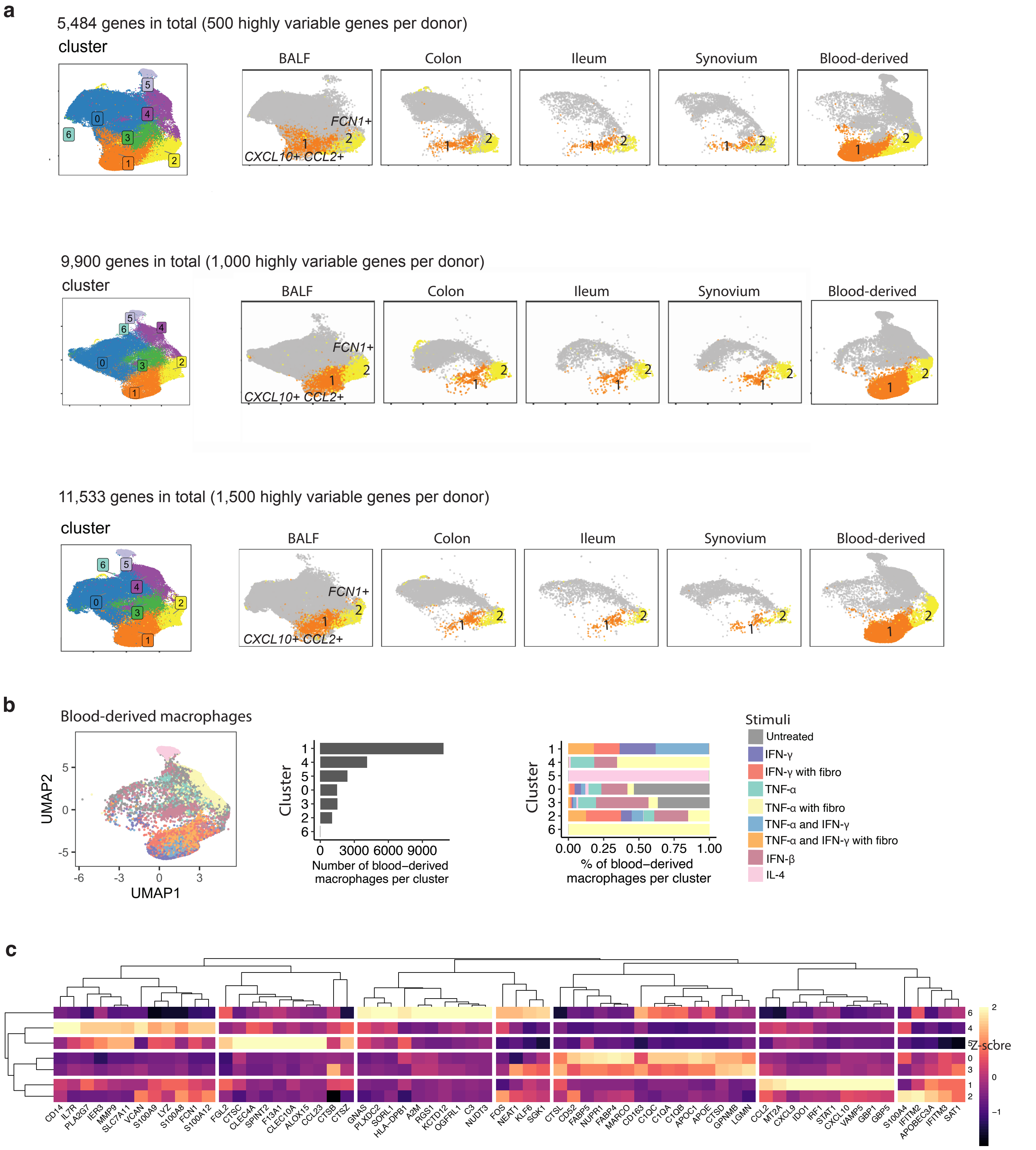

Fig S8. Integrative analysis of tissue-level macrophages and human blood-derived macrophages.
a. We examined the effect of the number of selected variable genes in the integrative analysis and clustering. We tested using top 500, 1,000, and 1,500 most highly variable genes within each donor, respectively. Cluster assignments are labeled and colored. The *CXCL10+ CCL2+* and *FCN1+* clusters are highlighted. **b**. For the blood-derived macrophages, we label each cell using the applied stimulatory condition. We also show the percent and exact number of stimulated blood-derived macrophages for each stimulatory condition and each cluster. **c**. Heatmap illustrating Z-score of the normalized gene expression of cluster-specific genes.

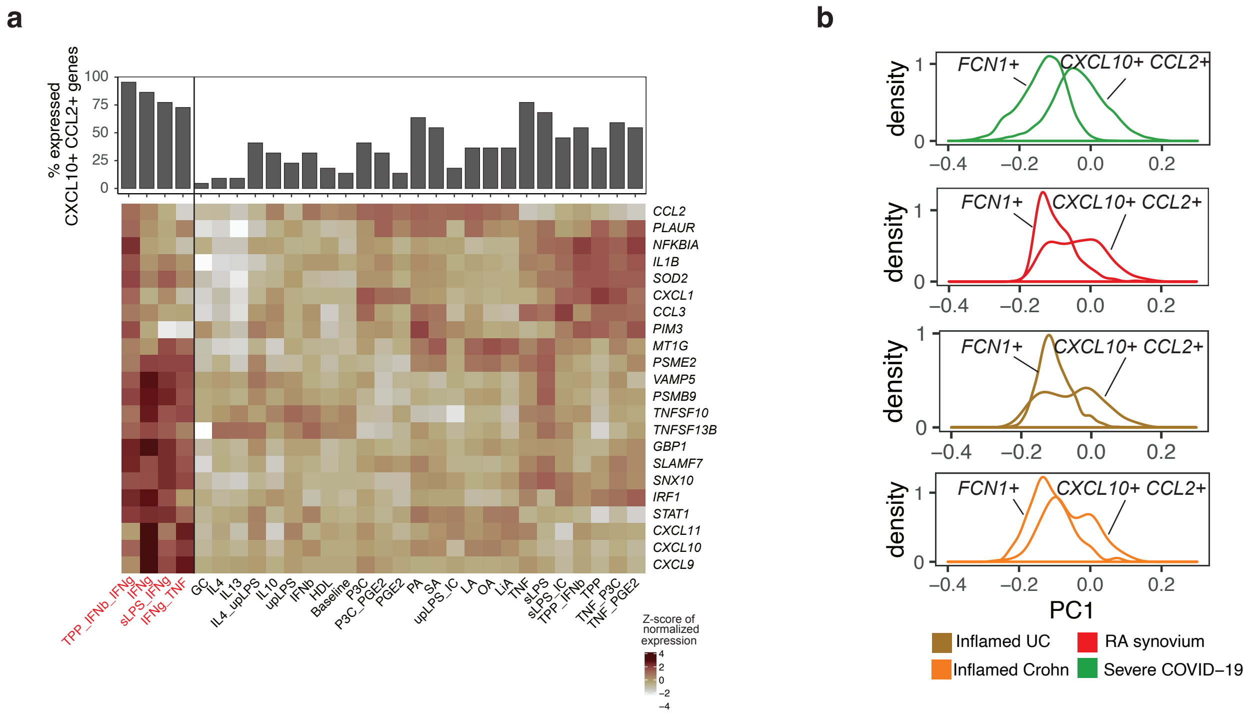

Fig S9. Assessment of previously reported stimulated macrophage spectrum analysis and alignment of macrophages from different disease tissues to a trajectory.
a. Z-score of normalized expression of *CXCL10*+ *CCL2*+ associated genes in stimulated macrophage spectrum analysis with 28 unique stimulatory conditions based on microarray (Xue, *et al*, 2014). We show percent of highly expressed *CXCL10*+ *CCL2*+ genes for each stimulatory condition in the barplot. **b**. Distribution of macrophages from each disease tissue aligned to PC1, which was identified by performing PCA analysis on the inflammatory macrophages. PC1 reflects a gradient from the *FCN1*+ state to the *CXCL10*+ *CCL2*+ state.
